# Association between internet use and modern contraceptive uptake among women of reproductive age in Rwanda: evidence from the 2019–2020 Rwanda DHS

**DOI:** 10.1186/s40834-026-00471-x

**Published:** 2026-08-08

**Authors:** Samuel Ndayishimye, Gbenga Olorunfemi, Bonfils Nahayo, Adesina Oladokun, Marie Francoise Mukanyangezi

**Affiliations:** 1 Reproductive Health Science Programme, Pan African University Life and Earth Sciences Institute (Including Health and Agriculture)- PAULESI. University of Ibadan, Ibadan, Oyo State Nigeria; 2 Department of Biomedical Sciences, University of Johannesburg, Johannesburg, South Africa; 3 Department of Obstetrics and Gynecology, University College Hospital, University of Ibadan, Ibadan, Nigeria; 4 Department of Clinical Pharmacy and Pharmacy Practices, School of medicine and Pharmacy, College of Medicine and Health Sciences, University of Rwanda, Butare, Rwanda; 5 Université Sagesse d’Afrique, Bujumbura, Burundi

**Keywords:** Internet, Modern contraceptive, Rwanda, Women of reproductive age

## Abstract

**Introduction:**

Modern contraceptives are pertinent to the reduction of unintended pregnancies, unsafe abortion, and maternal mortality. Unfortunately, the prevalence rate of modern contraceptive use is low in most Sub-Saharan African (SSA) countries including Rwanda. The use of internet promotes access to information on the need for childbearing postponement, prevention of unintended pregnancy, child spacing, and limiting family size. However, there is dearth of knowledge on the link between internet use and modern contraceptive use among Rwandan women. This study was therefore designed to fill the gap by assessing the relationship between exposure to the internet and uptake of modern contraceptives in Rwanda.

**Methodology:**

This cross-sectional design study was a secondary data analysis of the Rwanda Demographic and Health Survey. Sexually active women aged 15–49 years (*n* = 7290) were included in the study. The outcome variable and key independent variable was modern contraceptive use and internet use respectively. Data were analyzed using descriptive statistics, Chi-square test, and logistic regression model (α_0.05_).

**Results:**

Modern contraceptive prevalence rate was 58.8%, and 10.9% of the women were using internet services. Adjusting for other factors revealed that women who used internet had about 16% (AOR: 1.16, 95% CI: 0.93–1.45) increased odds of modern contraceptive use compared to women who did not. Other predictors of modern contraceptive use included age, marital status, educational level, religion, occupation, and the number of living children.

**Conclusion:**

Internet usage increased the utilization of modern contraceptive among Rwandan women of reproductive age. Thus, strategies to improve the use of modern contraceptive among Rwanda women of reproductive age should include providing appropriate easily accessible internet information about modern contraceptives. Government investment in appropriate social marketing of modern contraceptive through internet services is strongly recommended.

## Introduction

The utilization of modern contraceptives empowers individuals to make informed decisions, provide reproductive autonomy, and afford women the opportunity to control their childbearing behavior [1, 2]. Increased uptake of modern contraceptives significantly reduces the occurrence of unintended pregnancies. This reduction correlates with a decline in maternal mortality and morbidity rates and the incidence of unsafe abortions [3–5]. The uptake of modern contraceptives is low in Sub-Saharan Africa (SSA) with only 3 out of every 10 women reported as current users [6]. Sociodemographic and reproductive characteristics (such as area of residence, beliefs, educational level, number of living children, marital status, fearing of side effects and household wealth quintile, contribute significantly to the low uptake of modern contraceptives in the region); side effects; inappropriate information and poor health system have been reported as leading factors affecting modern contraceptive uptake in SSA [7–11]. Literature is consistent on the fact that low uptake of modern contraceptives in SSA is associated with high rates of unintended pregnancies, unsafe abortions, maternal mortality, and morbidity. For example, about 6.2 million cases of unsafe abortions and 38,000 maternal deaths due to clandestine abortions were reported annually in the SSA region [12]. These alarming figures could be significantly reduced through the adoption and utilization of modern contraceptives.

Rwanda has made significant advancements in its healthcare system over the years [13]. The healthcare infrastructure has been extensively reconstructed, aiming to improve access to essential healthcare services (including sexual and reproductive health services) among Rwandan citizens [14]. The prevalence of modern contraceptive among women of the reproductive age in Rwanda increased by 45% from 13% in 1994 to 58% in 2022 [15]. Despite the ongoing efforts, the prevalence of modern contraceptive use in Rwanda remains low [16]. The existing literature shows an increase in unintended pregnancies, teenage and adolescent motherhood, and the rise of new cases of sexually transmitted infections (STI) [17–19].

Similarly, there has been a rise in access to information communication and technology (ICT) infrastructures, leading to an increase in number internet users [20]. The current internet access among Rwandan population is about 24% [21]. Thus, access to ICT can potentially improve the access to appropriate sexual and reproductive health information, culminating in an increased uptake of modern contraceptive methods up to 53.8% [22]. Additionally, internet users can access information on modern contraceptives privately, without encountering long waiting times, distant travel, or potential stigma from certain healthcare providers and myths [23].

Studies that have documented the relationship between internet usage and modern contraceptive use in Rwanda is relatively scarce. Therefore, our study was to assess the relationship between internet use and modern contraceptive use among women of reproductive age in Rwanda using the 2019/2020 Rwanda Demographic and Health Survey data.

## Methodology

### Study area

Rwanda is situated in the Great Lakes region, sharing borders with Uganda to the North, Burundi to the South, Tanzania to the East, and the Democratic Republic of Congo (DRC) to the West. The majority of the population resides in rural areas, and the nation’s economy heavily relies on agriculture, particularly the exportation of tea and coffee [24, 25]. Rwanda is classified among the rapidly growing economies in Africa; However, approximately 37% of the national budget relies on foreign grants and debt.

### Study design and population

A cross-sectional design approach was used for data collection. The sample was nationally representative and involved 14,634 women of reproductive age (15–49 years). Thus, 7,290 women who were sexually active were included in the study.

### Source of data

This study utilized secondary data analysis from the Rwanda Demographic and Health Survey (RDHS), a comprehensive household survey representative of the national socio-demographic, health, and healthcare status. The survey was conducted between 2019 and 2020, capturing a dataset of five years ranging from 2016 to 2020. Data were collected by employing a two-stage cluster sampling method [26]. Primary clusters were randomly chosen from enumeration areas, and households were then randomly selected within these clusters. Subsequently, participants from each selected household were interviewed, contributing to the survey dataset.

### Dependent variable

In this study, the utilization of modern contraceptives was the outcome variable. Women who were using any modern contraceptive method (For example pills, intrauterine devices (IUDs), injections, male or female condoms, female sterilization, implants/Norplant, lactational amenorrhea, female condoms, emergency contraception, or the standard days method) were coded as 1. Those women using traditional methods (such as period abstinence, withdrawal, and other traditional approaches) and women not using any form of contraception were classified as 0. The categorization of modern contraceptive utilization is consistent with previous studies [27, 28].

### Independent variables

The utilization of the internet served as the primary independent variable, coded as ‘1’ for individuals who reported using the internet, while those women who never used the internet were coded as ‘0’. Additionally, the study included various socio-demographic characteristics including age, type of residence, highest educational level, marital status, religion, employment status, household wealth index, heard family planning information through media, and the number of living children. The age of women were classified as young women aged 15–24, mid-age women aged 25–34, and older women aged 35 and above [27]. Employment status was recategorized into ‘employed women’ and ‘not employed. Regarding the household wealth index, the initial five household wealth quintiles poorest, poor, middle, richer, and richest were collapsed into three categories: the ‘poor’ category comprising the poorest and poor categories, and the ‘rich’ category combining the richer and richest categories. Exposure to family planning information through media involved consolidating instances of women heard family planning messages through television, radio, or newspapers into one variable, Women who heard family planning information from at least one source were coded as having access and those who had not heard family planning were coded as no access to media. Finally, the number of living children was categorized into three groups: 0–2, 3–4, and 5 or more children.

### Data analysis

The statistical analysis was conducted using the Statistical Package for the Social Sciences version 25 (SPSS 25, IBM, USA). Univariable, Chi-square test, and multivariable analysis using logistic regression were performed employing a stepwise backward elimination method to determine both crude and adjusted odds ratios. Variables with a P-value < 0.05 in the bivariable analysis were included in the multivariable model, which was then assessed for goodness of fit using the Hosmer-Lemeshow test. The statistical significance level was set at a 95% confidence interval for a two-tailed test of hypothesis.

## Results

The overall prevalence of modern contraceptive uptake among women of reproductive age was 58.8%(*n* = 4287). Notably, older women aged 35 years and more accounted for half of the participants, while approximately two-thirds of study participants were married (*n* = 4706). A majority of the participants only had primary education. Nearly half of the study participants were identified as Protestants (46.9%) and 39.4% were from poor household wealth quintile. Only about one-fifth of the participants resided in urban areas, while 84.0% of total women were employed. Regarding number of children, 42.2% of participants had between 0 and 2 children. In all, 56% of participants had accessed media, while only 10.9% had used the internet. These baseline characteristics are summarized in Table 1.

**Table 1:** Socio-Demographic characteristics of the study participants

Study characteristic		Frequency (*n* = 7290)	Percentage (%)
Age	15–24	789	10.8
	25–34	2876	39.5
	35 and more	3625	49.7
Marital status	Married	4706	64.6
	Not married	2584	35.4
Highest education level	No education	922	12.6
	Primary	4770	65.4
	Secondary	1204	16.5
	Higher	394	5.4
Religion	Catholic	2660	36.5
	Protestant	3421	46.9
	Adventist	957	13.1
	others	252	3.5
Household wealth quintile	Poor	2863	39.3
	Middle	1453	19.9
	Rich	2974	40.8
Type of residence	Urban	1552	21.3
	Rural	5738	78.7
Respondent occupation	Not working	1165	16.0
	Working	6125	84.0
Number of living children	0–2	3079	42.2
	3–4	2583	35.4
	5 or more	1628	22.3
Access on Media	No	3208	44.0
	Yes	4082	56.0
Use of Internet	No	6498	89.1
	Yes	792	10.9

The chi-square test findings presented in Table 2, shows the factors linked to the uptake of modern contraceptives in Rwanda. It was observed that women cohabiting with partners showed a higher prevalence of modern contraceptive use compared to those who were married. The prevalence of modern contraceptive was more than 60% among younger women (15–24 years) (61.5%) and women aged 25–34 years (63.9%). While the prevalence of modern contraceptives was higher among women aged 35 years and older (53.8%). Factors such as educational level, internet usage, having occupation and having fewer than 5 living children were associated with the uptake of modern contraceptives. Women of the Catholic faith had the highest prevalence of modern contraceptive (62.0%).

**Table 2:** Prevalence of modern contraceptive uptake across the socio-demographic characteristics of the participants

Study characteristic		Modern contraceptive uptake		X^2^ / Fischer’	P-value
		Yes (%)	No (%)		
Age	15–24	485(61.5)	304(38.5)	70.2	< 0.001
	25–34	1838(63.9)	1038(36.1)		
	35–49	1951(53.8)	1674(46.2)		
Marital status	Married	2684(57.0)	2022(43.0)	13.9	< 0.001
	Not married	1590(61.5)	994(38.5)		
Highest education level	No education	473(51.3)	449 (48.7)	28.5	< 0.001
	Primary	2882(60.4)	1888(39.6)		
	Secondary	701(58.2)	503(41.8)		
	Higher	218(55.3)	176(44.7)		
Religion	Catholic	1650(62)	1010(38.0)	20.3	< 0.001
	Protestant	1931(56.4)	1490(43.6)		
	Adventist	546(57.1)	411(42.9)		
	others	147(58.3)	105(41.7)		
Household wealth quintile	Poor	1716(59.9)	1147(40.1)	9.49	0.08
	Middle	878(60.4)	575(39.6)		
	Rich	1680(56.5)	1294(43.5)		
Type of residence	Urban	890(57.3)	662(42.7)	1.33	0.257
	Rural	3384(59.0)	2354(41.0)		
Respondent occupation	Not working	635(54.5)	530(45.5)	9.72	0.002
	Working	3639(59.4)	2486(40.6)		
Number of living children	0–2	1711(55.6)	1368(44.4)	95.61	< 0.001
	3–4	1707(66.1)	876(33.9)		
	5 or more	856(52.6)	772(47.4)		
Access on Media	No	1883(58.7)	1325(41.3)	0.01	0.910
	Yes	2391(58.60	1691(41.4)		
Use of Internet	No	3842(59.1)	2656(40.9)	6.10	0.013
	Yes	432(54.5)	360(45.5)		

### Multivariable analysis

Table 3 shows factors related to modern contraceptive uptake among women of reproductive age in Rwanda. There was a significant association internet use and modern contraceptives uptake among sexually active women in Rwanda. The odds of modern contraceptive use among women who use the internet was about 16% higher than the odds of modern contraceptive uptake among women who did not use the internet (AOR: 1.16, 95% CI: 0.93–1.45). Age of participants, marital status, religion, respondents’ occupation and number of living children were significantly associated with modern contraceptive use. The odds of modern contraceptive use were two times higher among women aged 35 years and older as compared to women aged 24 years and younger. (AOR: 2.07, 95% CI: 1.68–2.55). Respondents who had secondary school education had nearly two folds odds of modern contraceptive uptake as compared to women who attended primary school. (AOR: 1.84, 95% CI: 1.12–3.01). Being a protestant (AOR: 1.31, 95% CI: 1,17-1.47), and a working woman (AOR: 1.24, 95% CI: 1.07–1.42) increased the odds of accepting modern contraceptive uptake compared to the catholic and non-working counterparts. On the other side, married women had about 14% lesser odds of using modern contraceptives (AOR: O.86, 95% CI: 0.76–0.97), while women who had 5 or more children had about 28% lesser odds of using modern contraceptive (AOR: 0.72, 95% CI: 0.62–0.83).

**Table 3:** Univariate and multivariable regression analysis showing factors associated with modern contraceptive uptake in Rwanda

Variables		Univariable analysis		^a^ Multivariable analysis	
		COR, [95% CI]	P-Value	AOR, [95% CI]	P-Value
Age	15–24	Ref		Ref	
	25–34	0.91, [0.77–1.06]	0.208	1.08, [0.90–1.29]	0.419
	35 and Above	1.37, [1.17–1.60]	< 0.001	2.07, [1.68–2.55]	< 0.001
Marital status	Not Married	Ref			
	Married	0.83, [0.75–0.92]	0.011	0.86, [0.76–0.97]	0.011
Highest Educational Level	No Education	Ref		Ref	
	Primary	1.76, [1.18–2.62]	0.006	1.05, [0.78–1.42]	0.749
	Secondary	1.24, [1.08–1.41]	0.002	1.84, [1.12–3.01]	0.015
	Higher	1.35, [1.06–1.62]	0.011	1.10, [0.84–1.46]	0.489
Religion	Catholic	Ref		Ref	
	Protestant	1.28, [1.15–1.44]	< 0.001	1.31, [1.17–1.47]	< 0.001
	Adventists	1.22, [1.04–1.44]	0.015	1.23, [1.04–1.45]	0.014
	Others	1.21, [0.91–1.60]	0.190	1.20, [0.90–1.60]	0.218
Respondent Occupation	Not Working	Ref			
	Working	1.24, [1.09–1.42]	0.002	1.42, [1.07–1.42]	0.003
Number of living Children	0–2	Ref		Ref	
	3–4	1.57, [1.32–1.87]	< 0.001	1.59, [1.33–1.89]	< 0.001
	5 or More	0.72, [0.62–0.83]	< 0.001	0.72, [0.62–0.83]	< 0.001
Use of Internet	No	Ref	Ref		
	Yes	1.21, [1.04–1.41]	0.014	1.16, [0.93–1.45]	0.018

^a^ Model adjusted for age, marital status, highest educational level, religion, respondent occupation, number of living children and internet use

## Discussions

Findings from this study, revealed that the prevalence of modern contraceptive use among women of reproductive age in Rwanda was 58.8%. Moreover, internet usage was associated with a 16% increase in the prevalence of modern contraceptive uptake. However, this finding is not peculiar to only Rwanda as previous studies conducted in Turkey, Ethiopia, and Indonesia similarly demonstrated that internet usage has positively influenced individuals’ choice towards accept and utilizing modern contraceptives [29–31]. In a study conducted across 8 Sub-Saharan African countries to assess the impact of internet usage on modern contraceptive uptake, it was found that the prevalence of modern contraceptive usage increased between 11.5% and 53.8% among those countries [22]. This increase in usage could be attributed to online platforms providing educational content about modern contraceptives. Access to such information enables women to privately access details without facing potential stigma from healthcare providers. Furthermore, it addresses the challenge of limited healthcare providers who may offer insufficient information regarding modern contraceptives. While to meet with healthcare providers require appointments for consultation, online content offers continuous accessibility, allowing individuals to access information at own convenience and from any location. This flexibility allows for repeated access, fostering better understanding and comprehensive learning about modern contraceptives. Nonetheless, inappropriate information on contraception may also be freely available online. Thus, government must make efforts to intentionally provide useful and correct information on contraception at accredited website so as for women of reproductive age to easily access the correct information.

Attending formal education plays a pivotal role in empowering women in securing jobs and make informed decisions about sexual and reproductive health (SRH) [32–34]. Evidently, as women gain higher levels of education, they tend to get jobs and become financially stable and independent. Thus, the more women become financially stable they are more likely to decide the number of children to have and when to have a child by using any form of modern contraceptive method.

The higher uptake of modern contraceptives among other religious groups compared to the Catholic Church members in Rwanda can be attributed to the conservative beliefs held within the Catholic Church. This is can be confirmed by the fact that health care facilities affiliated with these churches do not provide access to modern contraceptive methods [35, 36]. The number of living children influences the uptake of modern contraceptives, women having between 3 and 4 living children were 59% more inclined to use modern contraceptives compared to those with fewer children. This inclination seemingly aligns with the Rwanda national fertility rate, where the ideal number of children per women ranges between 3 and 4 [37]. While Rwandan culture condemn premarital and extra-marital sex, married sexually active women were less likely to uptake modern contraceptive. This can be attributed to the culture, women cohabiting with men are proudly to bear children since there are no stigmatization from the society [35].

### Strength and limitations

The DHS dataset is nationally representative and employs a robust sampling method, providing a strong basis for deriving conclusions. However, the data was not specifically collected to examine the relationship between internet usage and modern contraceptive utilization. Therefore, the dataset might contain biases or incomplete information that could affect the accuracy of the findings.

## Conclusion

The association between internet usage and modern contraceptive use among women of reproductive age in Rwanda is evident. However, while promoting modern contraceptive use, it is important to acknowledge socio-economic factors linked to its utilization. Additionally, educating women is crucial as it significantly contributes to improving the overall well-being of society. Government should improve the provision of relevant sexual and reproductive health information on the internet to further improve uptake of contraception’.

## Data Availability

Data are available upon request from the Demographic and Health Surveys portal: (https://dhsprogram.com/data/available-datasets.cfm).

## Abbreviations

AOR: Adjusted Odds Ratio
CI: Confidential Interval
COR: Crude Odds Ratio
DHS: Demographic and Health Survey
DRC: Democratic Republic of Congo
ICT: Information and Communications Technology
SRH: Sexual and reproductive health
SSA: Sub-Sahara Africa
STI: Sexually transmitted infections
